# Immunoregulation and clinical significance of neutrophils/NETs-ANGPT2 in tumor microenvironment of gastric cancer

**DOI:** 10.3389/fimmu.2022.1010434

**Published:** 2022-09-12

**Authors:** Shifeng Yang, Xiaoming Zou, Jiacheng Li, Hao Yang, Ange Zhang, Yanli Zhu, Lei Zhu, Lisha Zhang

**Affiliations:** ^1^ Department of Gastrointestinal Surgery, The Second Affiliated Hospital of Harbin Medical University, Harbin, China; ^2^ The Key Laboratory of Myocardial Ischemia, Ministry of Education, Harbin, China; ^3^ Department of General Surgery, The First Affiliated Hospital of Jiamusi University, Jiamusi, China; ^4^ Key laboratory of Microecology-immune Regulatory Network and Related Diseases School of Basic Medicine, Jiamusi University, Jiamusi, China

**Keywords:** gastric cancer, human umbilical vein endothelial cells, neutrophil extracellular traps (NETs), ANGPT2, tumor microenvironment

## Abstract

Although significant progress has been made in the study of gastric cancer (GC), clinicians lack reliable protein markers for accurate diagnosis and tumor stratification. Neutrophil extracellular traps (NETs) are networks of extracellular fibers composed of DNA from neutrophils. We have previously reported that abundant NETs are deposited in GC, damaging human umbilical vein endothelial cells (HUVECs) and triggering the release of tissue factors, leading to a hypercoagulable state in GC. However, the specific effects of NETs on HUVECs are unclear. We aimed to explore the functional changes caused by NETs on HUVECs, providing evidence that NETs may fuel GC progression. Through quantitative proteomics, we identified 6182 differentially expressed proteins in NET-stimulated HUVECs by TMT. The reliability of the TMT technique was confirmed by parallel reaction monitoring (PRM) analysis of 17 differentially expressed proteins. Through bioinformatics analysis, we found that NETs upregulate ANGPT2 in HUVECs. We comprehensively analyzed the prognosis, biological function, immune response, and therapeutic value of ANGPT2 in GC. We found that overexpression of ANGPT2 in GC is associated with poor prognosis and potentially regulates multiple biological functions. At the same time, ANGPT2 also predicted immunotherapeutic and chemotherapeutic responses in GC. In conclusion, NETs promoted ANGPT2 overexpression in the GC microenvironment. In the future, the neutrophil/NETs-ANGPT2 axis may provide a new target for the treatment of GC.

## Introduction

Gastric cancer (GC), one of the most common digestive tract malignant tumors, severely threatens human health worldwide, with high morbidity and mortality rates (1, 2). GC has strong heterogeneity and rapid progression, with the liver being the most common organ involved in hematogenous metastasis (3). The five-year survival rate of patients with GC after liver metastasis is less than 20% (4). New tumor markers, treatment targets, and therapeutic strategies are urgently required to improve the prognosis and the postoperative survival time of patients with GC.

The importance of the tumor microenvironment (TME) in tumorigenesis is being extensively explored (5, 6). GC is a dynamic entity. There is continuous communication between GC cells and their surrounding environment, which fuels and maintains phenotypic and heterogeneous factors of cancer cells (7, 8). The complex TME of GC includes fibroblasts, immune cells, adipocytes, vascular endothelial cells, and extracellular matrix (9). Tumor-associated neutrophils (TANs) are important matrix partners in carcinogenesis. Neutrophils can increase gastric cancer cell migration and invasion abilities (10–12). Neutrophil extracellular traps (NETs) are defensive mechanisms deployed by neutrophils (13, 14). Current studies suggest that NETs may exert double-edge effects on tumors, possibly due to the physicochemical properties of NETs (15, 16). NETs are DNA-based network structures rich in a variety of active proteins (17, 18). NETs can capture circulating tumor cells through DNA reticular structures. Cools-Lartigue et al. have shown that NETs can prevent circulating tumor cells from entering the vascular system of the lung and liver, suggesting that NETs are closely related to tumor progression (19).

Although endothelial cells are relatively stable cells in the human body, they can re-enter the cell cycle under certain triggering conditions (20). Some studies have shown that vascular occlusion and blood hypercoagulability adversely affect the hemodynamic stability of patients, possibly increasing the number of circulating tumor cells captured by NETs in tumor patients (21–23). However, NETs can damage endothelial cells, and captured tumor cells can spread, forming new metastatic foci after adhering to activated endothelial cells (15). Therefore, clarifying the mechanism between NETs and endothelial cells is pivotal to explaining tumor occurrence and development. In this study, we found that NETs act on HUVECs, triggering the release of coagulation-related factors and promoting tube formation. Therefore, the interaction between NETs and HUVECs is worth exploring.

In our study, we used proteomic technology to characterize differentially expressed proteins in NET-stimulated HUVECs. Parallel reaction monitoring (PRM) analysis was used to validate 17 selected proteins. Finally, ANGPT2 was chosen as the target protein. ANGPT2 belongs to the angiopoietin (Ang) family (24). ANGPT2 expression plays a vital role in vascular remodeling and inflammation (25). As an early autocrine initiator of angiogenesis, ANGPT2 first destabilizes resting blood vessels, thus allowing VEGF to drive the proliferation and chemotactic migration of vascular buds. ANGPT2 also activates TIE2-expressing monocytes/macrophages (TEM), which promote angiogenesis, tumor formation, metastasis, and immunosuppression (26). ANGPT2 regulates vascular remodeling and tumor progression under many pathological conditions through different effects on TIE2 signaling. Targeting the ANG-TIE2 axis can significantly improve the effect of tumor immunotherapy (27). Therefore, it also proves that ANGPT2 participates in tumor immune response and targeting neutrophil/NETs-ANGPT2 may be a new target of anti-tumor therapy in the future.

## Materials and methods

### Patients and tissue samples

This study included 20 healthy participants and 60 patients with newly diagnosed primary gastric cancer by pathological examination who were admitted to the Second Affiliated Hospital of Harbin Medical University from October 2019 to April 2021. We based the evaluation (TNM), staging, and histological classification of gastric cancer on the 8th edition of the American Joint Commission on Cancer (34). The inclusion criterium was patients aged 18–65 years without any endocrine, cardiovascular, hematological, and infectious diseases. The exclusion criteria were as follows: patients who are not pregnant, without coexisting cancers, and those who did not receive any antineoplastic treatment before surgery. Samples of cancerous and para-cancerous tissues of patients with gastric cancer were collected. All patients provided informed consent, and this study was approved by the Internal Audit and Ethics Committee of the Second Affiliated Hospital of Harbin Medical University. Ethical review approval document No : KY2021-075.

### Isolation of neutrophils

A neutrophil isolation kit (TBD sciences, Tianjin, China) was used to isolate neutrophils from blood samples of patients with GC or healthy controls. Venous blood samples were collected using a 5 ml catheter containing 3.2% sodium citrate. Then, a 5 mL neutrophil separation solution was mixed with anticoagulants and whole blood. The neutrophil layer was added to the erythrocyte separation solution and centrifuged repeatedly at 450 ×g for 5 min at 24°C until red blood cells disappeared. Neutrophils were resuspended in 1 mL PBS and counted.

### Generation, isolation, and preparation of NETs

Purified neutrophils (1 × 10^6^/well) were seeded in a 6-well plate, stimulated with 100 nM PMA, and cultured at 37°C, 5% CO_2_, for 4 h. Then, the medium was gently removed, leaving NETs and neutrophils attached to the bottom. Pre-cooled PBS without calcium and magnesium was added to elute NETs and neutrophils. Liquid samples were collected in 6-well plates, centrifuged at 450 ×g, 4°C, for 10 min, and the supernatant was collected. The collected supernatant was centrifuged at 4°C 15000 ×g for 15 min. The concentration of NETs was determined using a micro-DNA instrument, and samples were stored at -20°C.

### Cell culture

Primary human umbilical vein endothelial cells (HUVECs) were purchased from PROCELL (Wuhan, China). All cell lines were verified by a short tandem repeat (STR) map and negative detection of mycoplasma contamination. All cell lines were cultured at 37°C in a 5% CO_2_ humidified laboratory and cultured in ECM (Scien Cell, USA) medium containing 10% fetal bovine serum (FBS) for 5 days; then, the medium was replaced with FBS-free medium.

### Stimulation of HUVECs

HUVECs were cultured in a medium containing NETs (NETs were derived from neutrophils from patients with GC, as described above) at 37°C, 5% CO_2_, for 24 h. To conduct the inhibition test, isolated NETs were pretreated with DNase-1 (100 U/mL) at 37°C for 1 h, and then co-cultured with HUVECs. At a specific time, point, HUVECs were collected by centrifugation for follow-up experiments, including western blot, after protein extraction and measurement of target protein expression.

### Tube forming experiment

The Corning matrix glue was diluted with matrix glue at a 1:1 ratio in a 24-well plate, with 200 μl per well. The mix was incubated at 37°C for 30 min to obtain a gelatinous base glue. HUVECs were digested, and a single-cell suspension was prepared. The conditioned medium was replaced by FBS-free medium. Next, 20 000 cells were seeded in each well. The process of tubule formation was observed under a microscope at 37°C for 2–4 h.

### Immunofluorescence

HUVECs were stimulated with 0.4 µg/mL NETs prepared hours in advance, and then an immunofluorescence experiment was carried out. The medium in the 24-well plate was removed, cells were fixed in 1% PFA for 15 min, and washed twice with PBS. Samples were then incubated in 3% BSA for 30 min, washed twice with PBS, and incubated overnight at 4°C with primary antibodies: diluted anti-VWF (Abcam), anti-ANGPT2 (Affinity) (13% BSA 500). Specimens were washed twice with PBS and incubated with secondary antibodies: diluted AF-488 or AF-594 antibody for 30 min. For nuclear staining, cells were incubated with DAPI for 10 min and washed with PBS twice. Sharp tweezers were used to remove adherent cells from each well and deposit them with the cellular side upside down on a slide coated with anti-quenched glycerol. Specimens were observed and imaged under a confocal microscope. All results were expressed as mean ± standard deviation (SD). SPSS or GraphPad Prism 8.0 were used to analyze all data. Multiple groups were compared and analyzed by one-way. ANOVA. Two pairs variables were analyzed by paired t-test. Statistical significance was determined when P< 0.05.

### TMT-MS/MS labeled

The trypsin-hydrolyzed peptides were desalted with StrataXC18 (Phenomenex) and then freeze-dried in a vacuum. Peptides were dissolved with 0.5 M TEAB and labeled according to the operating instructions of the TMT kit. Briefly, after the labeled reagent is thawed, it is dissolved with acetonitrile, mixed with the peptide, and incubated at room temperature for 2 h; the labeled peptide is mixed, desalted, and vacuum freeze-dried.

### Quantitative analysis

The results of database search of MS data show the signal intensity value (Intensity) of each peptide in different samples. According to this information, the relative quantity of protein is calculated by the following steps: First of all, the signal intensity values (I) of peptides in different samples were centralized, and the relative quantitative values (R) of peptides in different samples were obtained. The formula is as follows: where i represents the sample and j represents the peptide:


Rij = Iij/Mean(Ij)

The relative quantitative value of protein was calculated. The relative quantitative value of protein was expressed by the median of the relative quantitative value of specific peptide corresponding to protein. The formula is as follows: where k represents protein. J represents the specific peptide to which the protein belongs:




Rik = Median (NRij,j∈k)

### Differential protein screening.

Protein difference analysis first selects the sample pairs that need to be compared, and takes the ratio of the mean value of all biological repetitive quantitative values of each protein in the comparison sample pair as the difference multiple (Fold Change, FC). For example, the multiple of protein difference between sample group A and sample group B is calculated. The calculation formula is as follows: R represents relative quantity of protein, i represents sample, k represents protein:


$$FC_{A/B,k}=Mean(R_{ik},i\in A)/Mean(R_{ik},i\in B)$$

According to the above difference analysis, when P value ≤0.05, the change threshold of differential expression was more than 1.3 as the threshold of significant up-regulation, and less than 1.3 as the threshold of significant down-regulation.

### Establishment of animal model

Animal experiments were carried out at the Animal Experimental Center of the Key Laboratory of Myocardial Ischemia of the Second Affiliated Hospital of Harbin Medical University in strict accordance with the scheme approved by the Animal Care and Use Committee. Ten BALB/c nude mice aged 5–7 weeks were purchased from Weitong Lihua Experimental Animal Technology Co. Ltd and kept in a 22°C aseptic animal house. Food and autoclaved water were provided. The mice were randomly divided into the control (n = 5) and DNAse-1 treatment (n = 5) groups. All mice were anesthetized with 2% isoflurane mixture; the axillary skin of the mice was disinfected with sterilized cotton balls; 1 × 106 HGC-27 gastric cancer cells were subcutaneously injected into the armpit; and cotton swabs were applied to stop the bleeding. The mice in the treatment group were intraperitoneally injected with deoxyribonuclease (DNase-I, 50μg/mouse, Roche) every 12 h until they were euthanized. The tumor volume in each mouse was measured once every 3 days and calculated using the following formula: 0.5 × length × width2. Euthanasia was performed on all mice after 18 days. The tumor tissues were harvested, weighed, soaked in 4% paraformaldehyde for 24 h, embedded in paraffin, and divided into 4-μm paraffin sections for follow-up immunohistochemical staining.

### Screening of ANGPT2 genes

NET+ vs. Control difference analysis was performed with a cutoff of P-value< 0.05. 123differentially upregulated proteins were identified. We then used machine learning algorithms, svm-rfe and random Forest-rfe, to reduce dimensionality, yielding 29 proteins. Then we selected the 17 most valuable proteins according to the literature.

### STAD dataset and preprocessing

STAD-related data and corresponding clinical information were downloaded and collected from TCGA (https://xenabrowser.net/). The transcriptional spectrum of 414 patients with STAD cancer was obtained from TCGA, of which 388 patients with STAD had complete OS information. 36 paracancerous STAD samples were obtained from TCGA. The transcriptional data from 174 normal gastric samples were collected from the genotype-tissue expression project (GTEx; https://www.gtexportal.org). The fragment (FPKM) value per kilobase was converted to the transcript (TPM) value per kilobase.

### Carcinogenic characteristics of ANGPT2

The difference of ANGPT2 between GC samples and non-cancer samples was analyzed in TCGA and GTEx. We estimated the ANGPT2 of 388 patients in the TCGA-STAD dataset, then sorted patients into high ANGPT2 and low ANGPT2 groups according to the P-value of the best cutoff. Kaplan–Meier curves were used to analyze the association between OS and ANGPT2. Univariate and multivariate Cox regression analysis was performed for ANGPT2.

### Genome change

Somatic mutation data and somatic copy number variation (CNV) were collected from TCGA. Genomic identification (GISTIC) analysis of important targets in cancer is used to evaluate genomic characteristics. GISTIC2.0 analysis (https://gatk.broadinstitute.org) was used to evaluate the increase or loss of copy number of CNV landscape and amplification or deletion peaks.

### The immunological characteristics of TME

We used the Estimation of Stromal and Immune cells in Malignant Tumor tissues using Expression (ESTIMATE) algorithm to estimate the abundance of immune cells and the level of stromal cell infiltration. The level of immune infiltrating cells in STAD was analyzed comprehensively using Tumor Immune Estimation Resource2.0 (TIMER2.0; http://timer.cistrome.org/) network server. The relative proportion of ten kinds of immune cells in the tumor was estimated by the MCPcounter algorithm. The infiltration level of 28 kinds of immune cells was expressed as the enrichment fraction based on the corresponding characteristics using a single sample genome enrichment analysis (ssGSEA) using the GSVA R package. The response of STAD to immunotherapy was evaluated by a submap algorithm.

### Functional analysis

Gene set variation analysis (GSVA) was carried out with the GSVA R package.

### Prediction of drug response

Pharmacogenomics data from cancer drug sensitivity genomics (GDSC, https://www.cancerrxgene.org/) are used to predict the drug sensitivity of included cases. Drug sensitivity was calculated with the oncoPredict R package.

### Statistical analysis

Wilcoxon test was used to compare non-normally distributed data. The t-test was used to compare normally distributed variables between two groups. Using the R package survminer, the Kaplan–Meier survival graph was used to estimate the OS between two groups. The Cox regression of survival analysis was conducted with the R package survival. All heat maps were drawn with the R pheatmap package. The data were visualized with R ggplot2 package. P< 0.05 was considered statistically significant.

## Results

### 3.1 Gastric cancer tissue shows multiple blood vessels with abundant NETs deposition around blood vessels

**Figure 1:**
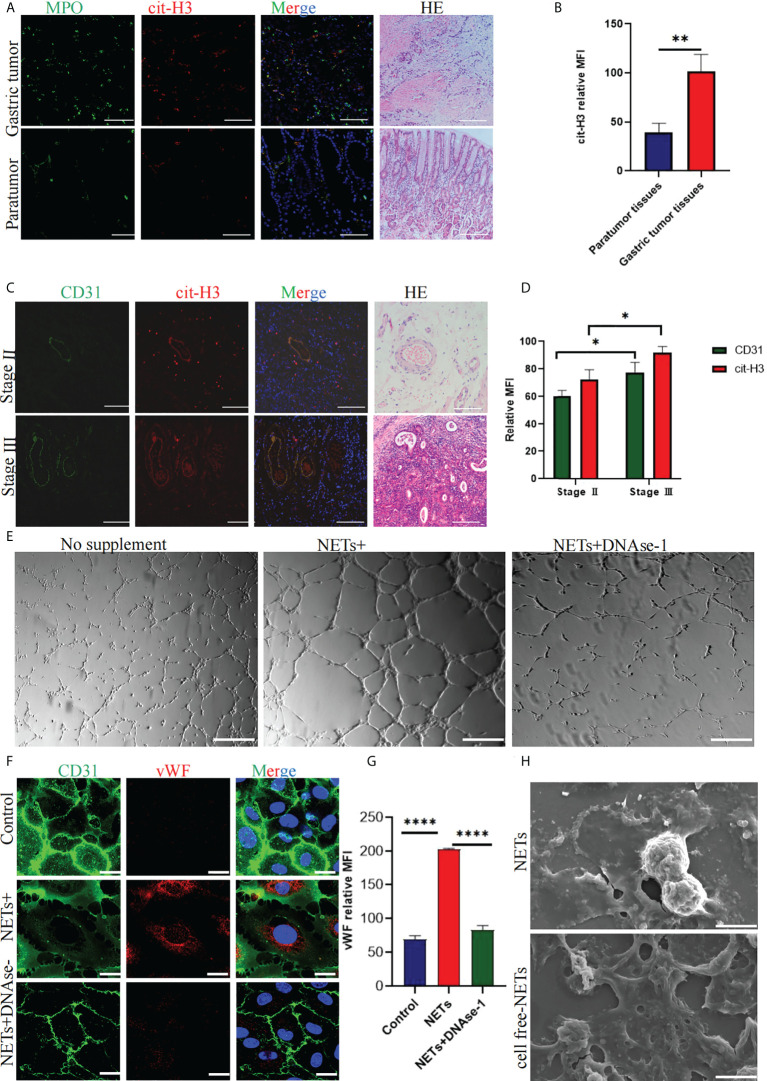
Expression of NETs in gastric cancer (GC) and paracancerous tissues and quantification of related factors in NET-stimulated HUVECs. **(A)** NETs deposition in GC and paracancerous tissues was analyzed by immunohistochemical staining. HE staining was used to analyze the distribution of microvessels in GC and paracancerous tissues. **(B)** Quantitative analysis of cit-H3 in GC and paracancerous tissues. **(C)** The localization of CD31 and NETs marker cit-H3 in GC. HE staining was used to analyze the distribution of microvessels in different stages of GC. **(D)** Mean fluorescence intensity of CD31 and cit-H3 in tumor tissues at different stages. **(E)** Tubule formation of HUVECs stimulated with PBS, NETs, and NETs+DNAse-1 was analyzed by a tube forming experiment. **(F, G)** The vWF expression on HUVECs was detected by confocal microscopy and analyzed with Image J software [the expression is indicated as the mean fluorescence intensity (MFI). **(H)** NETs and cell-free NETs imaged by scanning electron microscope. 20× objective. Scale bars indicate 20 mm. Arrows indicated ETs. All values are mean ± SD. ****P < 0.0001, **P < 0.01, *P < 0.05 by one -way ANOVA or t-test.

Immunofluorescence staining showed that GC tissue (n = 30) had more NET deposition (**Figures 1A, B**) than adjacent tissue (n=30) and staining of HE showed microvessels in GC; the worse the staging, the more abundant the microvessels (**Figures 1A, C**). CD31 immunofluorescence staining was used to characterize the blood vessels of GC, several NETs deposits were found around the vessels (**Figures 1C, D**).

### NETs enhance the angiogenesis ability of HUVECs and the expression of damage factor vWF

After stimulating HUVECs with 0.4 μg/ml NETs, more tubules (**Figure 1E**) were formed compared with the PBS group. Immunofluorescence staining showed that VWF expression and the intercellular space increased after HUVECs were stimulated with NETs (**Figure 1F**). DNAse-1 could attenuate the above phenomena (**Figures 1E, F**). **Figure 1G** shows the result of quantitative analysis of the mean fluorescence intensity of VWF, which is statistically significant. **Figure 1H** shows scanning electron microscope images of NETs and cell-free NETs.

### Identification of differentially expressed proteins in NET-stimulated HUVECs by TMT-MS/MS and PRM

**Figure 2:**
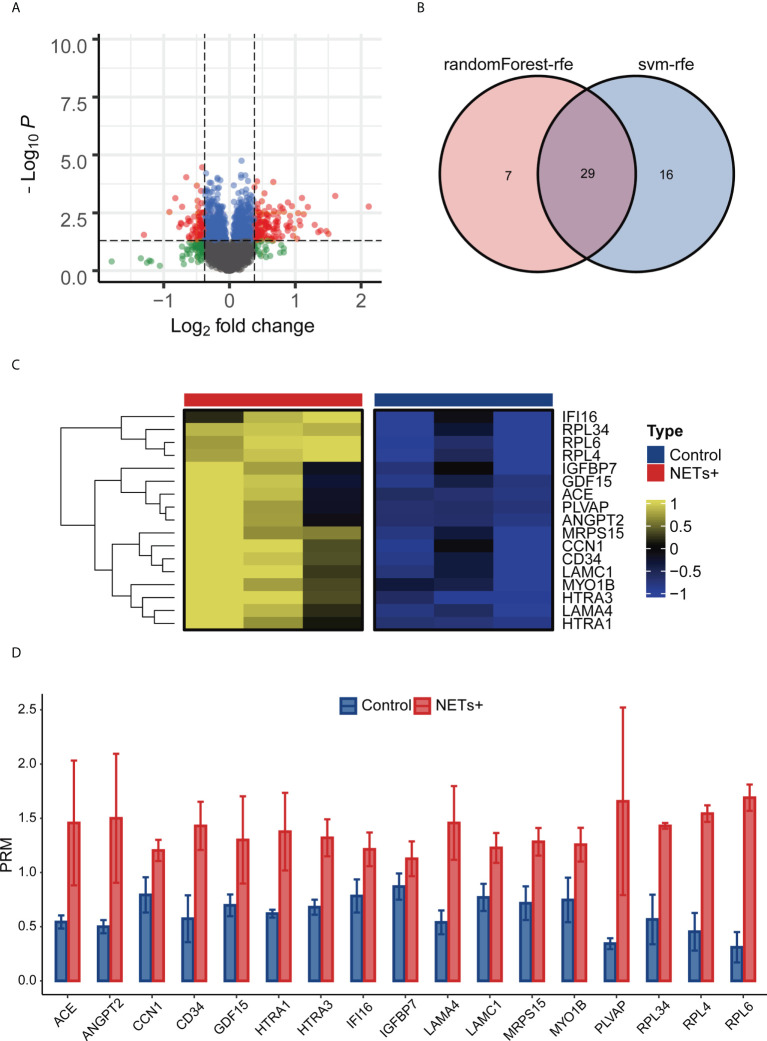
Screening ANGPT 2 based on TMT-MS/MS data and PRM analysis. **(A)** Volcanic map of the results of NETs + vs. Control difference analysis. **(B)** Svm-rfe and randomForest-rfe reduce dimensionality based on up-regulating differential proteins. **(C)** PRM analysis heat map of 17 differential proteins. **(D)** Analysis of bar plot by PRM of 17 differential proteins.

For TMT-MS/MS analysis of NET-stimulated and unstimulated HUVECs, a total of 363519 secondary mass spectra were collected, including 83412 effective mass spectra, with a utilization rate of 22.9%. Among quantifiable proteins, 123 upregulated and 73 downregulated proteins were observed in the NET-stimulated group but not in the unstimulated group (**Figure 2A**). Then the dimension of 123 upregulated differential proteins was reduced with machine learning, reduce the number of proteins to 29 (**Figure 2B**). By reviewing the literature related to tumor progression and angiogenesis, 17 proteins were selected from the above 29 proteins for PRM analysis, which, namely ACE, ANGPT2, CCN1, CD34, GDF15, HTRA1, HTRA3, IFI16, IGFBP7, LAMA4, LAMC1, MRPS15, MYO1B, PLVAP, RPL34, RPL4, and RPL6 (**Figures 2C, D**). The results showed that the expression of these proteins was significantly upregulated in the NETs-stimulated group.

### ANGPT2 is associated with poor prognosis of patients with gastric cancer and participates in carcinogenesis

**Figure 3:**
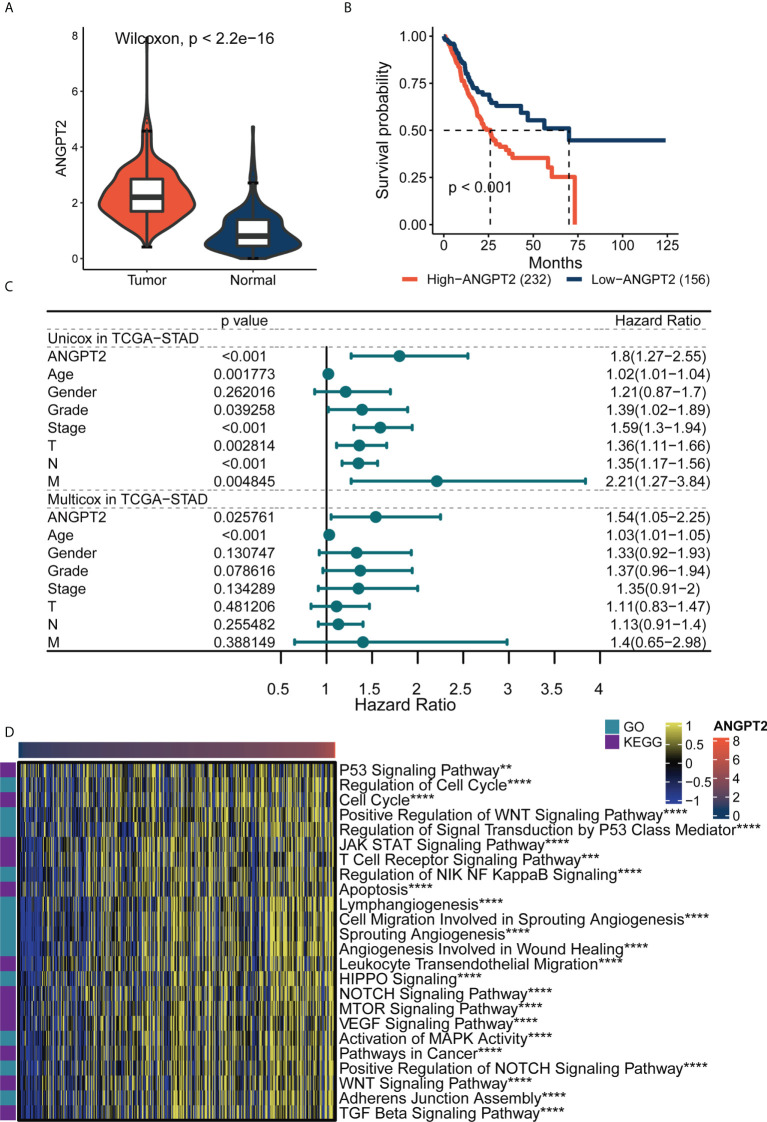
The importance of ANGPT2 gene in TCGA-STAD. **(A)** ANGPT2 expression level between STAD samples from TCGA cancer database and TCGA paracancerous plus GTEx normal samples. **(B)** Kaplan–Meier curves of high and low ANGPT2 groups in TCGA data. **(C)** Forest map of univariate and multivariate cox regression based on TCGA dataset and clinical variables. **(D)** GSVA thermomaps show functional pathways (GO and KEGG cancer-related pathways) significantly related to ANGPT2 in TCGA. **P < 0.01, ***P < 0.001 and ****P < 0.0001.

The TCGA database analysis indicated that ANGPT2 expression in GC tissues (414) was significantly higher than in para-cancerous (36) and normal tissues in GTEx (174) (**Figure 3A**). The Kaplan–Meier curve results showed overall svrvival (OS) that high ANGPT2 expression was associated with worse survival (**Figure 3B**). Univariate and multivariate analysis based on TCGA dataset revealed that ANGPT2 can work as an independent risk factor in GC. (**Figure 3C**). GSVA analysis highlighted that ANGPT2 was significantly associated with many important tumor-related pathways, such as the p53 signaling pathway, regulation of DNA damage response signal transduction, p53 class mediator, JAK-STAT signaling pathway, and cell migration involved in angiogenesis. We also observed that ANGPT2 participated in immune response-related processes, such as the T cell receptor signaling pathway and leukocyte trans endothelial migration (**Figure 3D**). Therefore, the high ANGPT2 expression can activate a variety of tumor-related pathways and immune responses, suggesting that ANGPT2 plays a significant role in the occurrence and development of GC.

### ANGPT2 expression and the diversity of genomic changes

**Figure 4:**
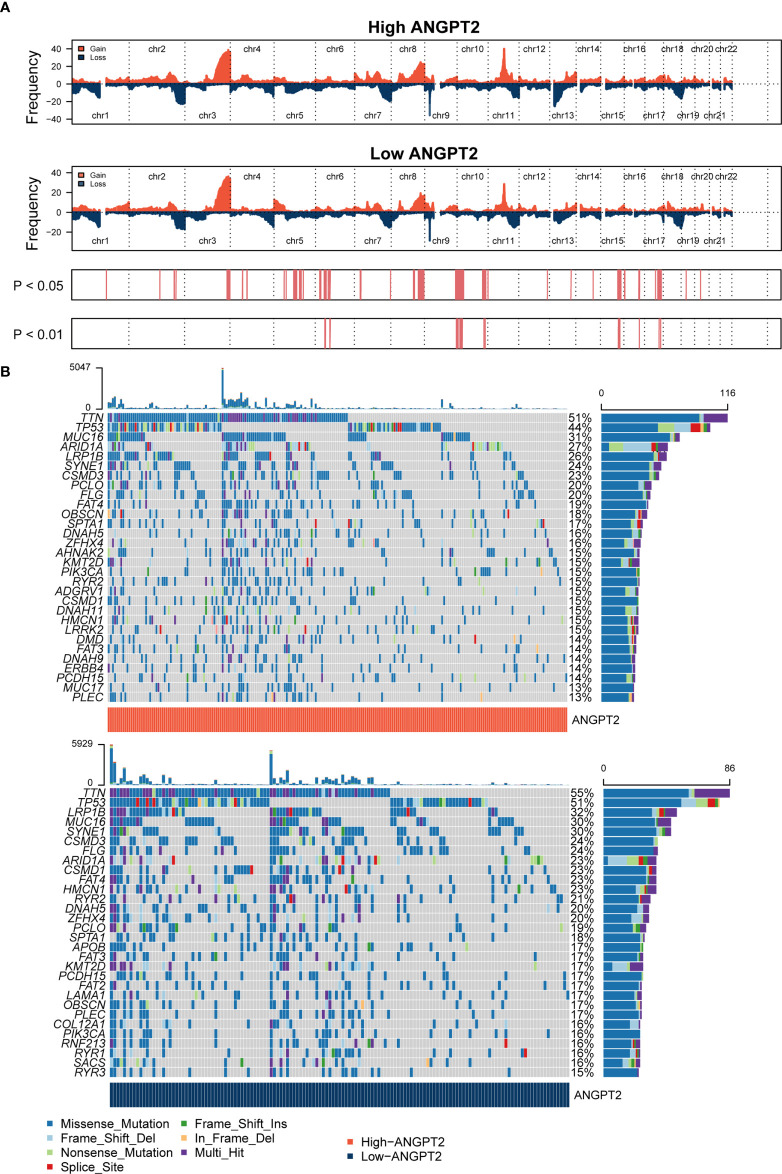
Genomic changes related to ANGPT2 in STAD samples **(A)** Changes in the copy number of ANGPT2 between high and low STAD groups. **(B)** Somatic mutation waterfall map in STAD of high and low ANGPT2 groups.

CNV in the human genome can affect gene expression by altering gene expression, destroying regulation or coding regions, or changing genome structure, ultimately affecting the normal function of genes. CNVs have been directly or indirectly related to various diseases. Cancer genomes are usually characterized by somatic CNV, often accompanied by amplification of proto-oncogenes or deletion of tumor suppressor genes. CNV and somatic mutation analysis were conducted in the TCGA GC data set. The results of the global CNV map showed that the amplification of the high ANGPT2 group was concentrated in Chr17, especially 17q12, and the deletion was concentrated in Chr4 and Chr16, especially loci 4q35.2 and 16q23.1. In the low ANGPT2 group, the increase of Chr17, the loss of Chr9, and the loss of Chr16 were identified. The amplification was mainly found in 17q12, and the deletion was mainly concentrated in 9p21.3 and 16q23.1 (**Figure 4A**, **Supplementary Figure S1**). The global view of mutation distribution shows that actin (TTN) and cell tumor antigen p53 (TP53) mutations are the most abundant in both the high ANGPT2 and low ANGPT2 groups, 51% and 44%, respectively (**Figure 4B**). The next three most common mutations in the high ANGPT2 group were MUC16 (31%), ARIDEA (27%), and LRP1B (26%). In the low ANGPT2 group, LRP1B (32%), MUC16 (30%), and SYNE1 (30%) (**Figure 4B**).

The mutation frequency ratio between the high ANGPT2 and low ANGPT2 groups was assessed by Fisher’s exact test and sorted by increasing P-values. The sudden change load of the high ANGPT2 group was lower than that of the low ANGPT2 group (**Supplementary Figure S2A**). At the same time, we analyzed the coexistence or mutually exclusive mutation (**Supplementary Figures S2B, C**) of the 25 most frequent mutations. In the high ANGPT2 group, TTN and CSMD1 mutations frequently coexisted. ARID1A and PIK3CA, SYNE1 and SPTA1, FAT4 and DMD were also closely related mutation sites. In the low ANGPT2 group, the common co-mutations were FLG, SACS, OBSCN, and PCLO. Moreover, we found that the changes of some gene pairs were mutually exclusive, such as TP53 and PIK3CA, TP53 and ARID1A in the high ANGPT2 group; TP53 and ARID1A in the low ANGPT2 group (**Supplementary Figures S2B, C**).

### Immune correlation of ANGPT2 in TCGA cohort

**Figure 5:**
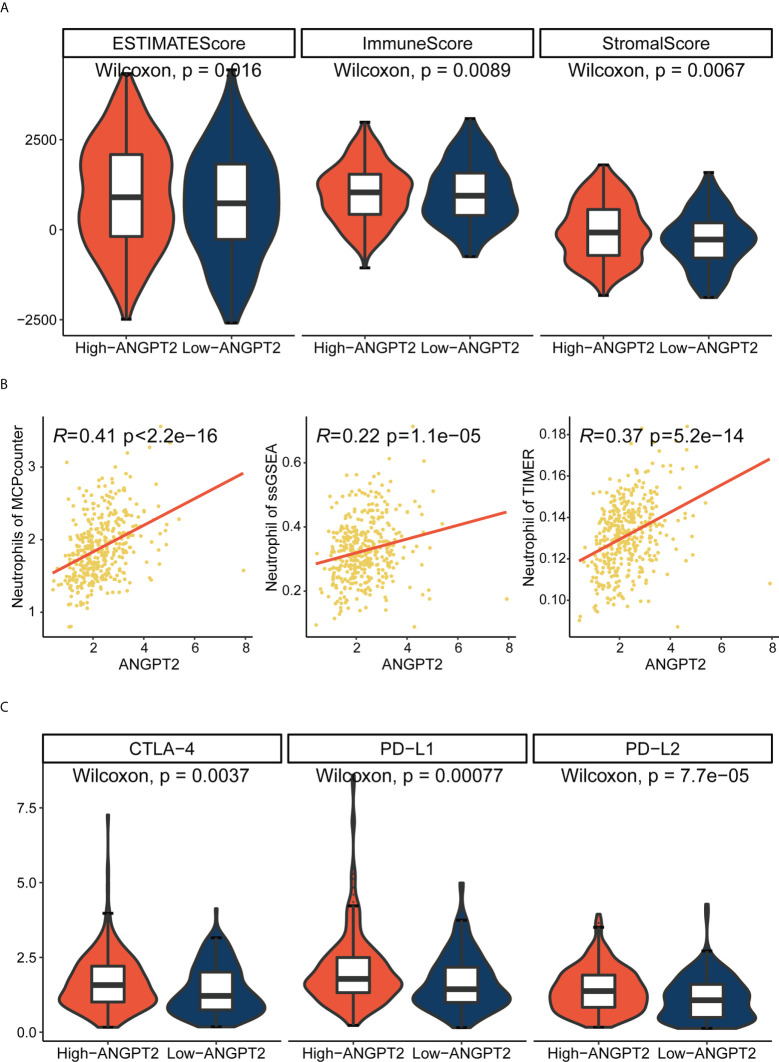
Relationship between ANGPT2 and immune characteristics in TCGA cohort. **(A)** Changes of ANGPT2 and ESTIMATE score, immune score, and stromal score. **(B)** Correlation between ANGPT2 and neutrophil cell infiltration level in three immune infiltration estimation algorithms. **(C)** The relationship between ANGPT2 and three classical immune checkpoints.

ESTIMATE analysis showed that the ANGPT2 immune and stromal scores were significantly increased (**Figure 5A**) in the high expression group, implying worse patient prognoses. We further analyzed the correlation between ANGPT2 expression and neutrophil infiltration. We found a positive correlation based on ESTIMATE, MCP counter, ssGSEA, and TIMER algorithms (**Figure 5B**), consistent with our results. Neutrophil infiltration correlates with NET deposition. At the same time, we also analyzed the correlation between ANGPT2 and other immune cell infiltration, and found that the high expression of ANGPT2 was significantly correlated with Macrophages M0 、NK cells resting and Mast cells activated (**Supplementary Figures S3A, B**). So we have reason to think that ANGPT2 is involved in the regulation of immune microenvironment for gastric cancer.

### Potential targets of immunotherapy and chemotherapy in patients with gastric cancer with high ANGPT2 expression

**Figure 6:**
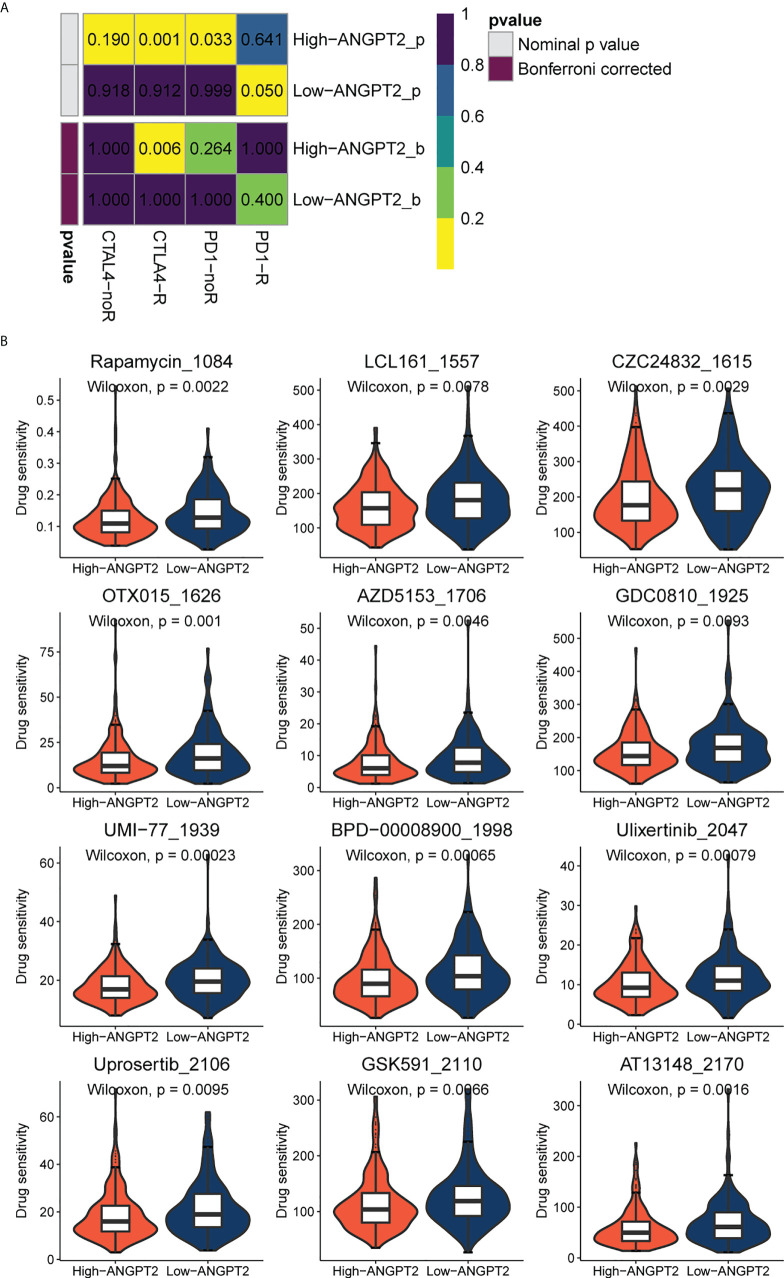
Immunotherapy and chemotherapy involved in ANGPT2 in TCGA-STAD. **(A)**Submap analysis of ANGPT2 levels in TCGA-STAD. **(B)** Box diagrams for estimating drug sensitivity of several GDSC chemotherapeutic drugs in the high ANGPT2 and low ANGPT2 groups.

Since immune checkpoint inhibitors are the basis of immunotherapy, immune checkpoint expression is of great significance in guiding clinical practice. The submap result showed that the response to immunotherapy differed between the high ANGPT2 and the low ANGPT2 groups. The result indicated that GC patients with high expression of ANGPT2 are more likely to respond with immunotherapy (**Figure 6A**), which is a significant reference in accurately choosing immunotherapy.

We analyzed the correlation between ANGPT2 and multiple immune checkpoints. The results showed that ANGPT2 was closely related to the level of multiple immune checkpoints in GC, PD-L1, PD-L2, and CTLA-4 (**Figure 5C**) are the most significant. These results suggest that inhibition of ANGPT2JI may be beneficial to immunotherapy.

We used the GDSC database to evaluate chemotherapeutic drug responses in high ANGPT2 and low ANGPT2 groups and measured accuracy by 10x cross-validation. The results showed the sensitivity of the high expression group to the following chemotherapeutic drugs, Rapamycin, LCL161, CZC24832, OTX015, AZD5153, GDC0810, UMI-77, BPD-00008900, Ulixertinib, Uprosertib, GSK591, and AT13148 was lower relative to the low expression group (**Figure 6B**). Therefore, patients with low ANGPT2 expression might experience better chemotherapeutic outcomes, and the efficacy of these chemotherapeutic drugs is worthy of further exploration.

### ANGPT2 is highly expressed in gastric cancer, and NETs promote ANGPT2 expression in HUVECs

**Figure 7:**
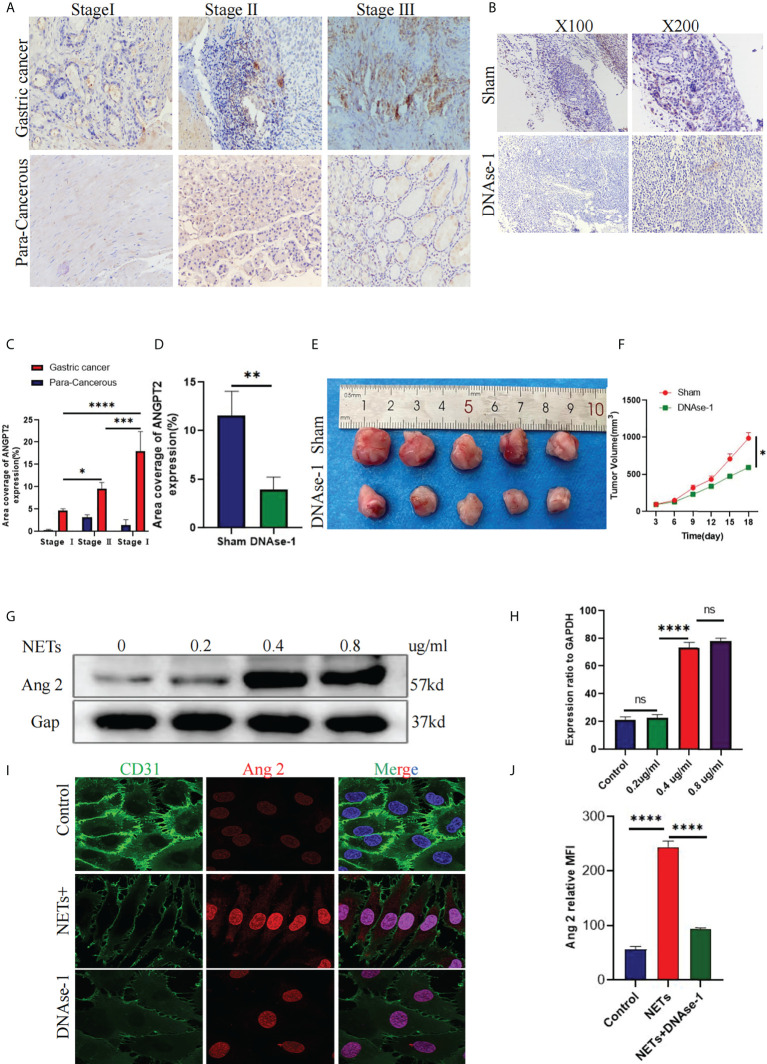
ANGPT2 expression in NET-stimulated HUVECs. **(A)** Immunohistochemical analysis of ANGPT2 expression in gastric cancer and paracancerous tissues of different stages. **(B)** ANGPT2 expression in mouse subcutaneous tumor tissue was analyzed by immunohistochemical staining. **(C)** Quantitative analysis of ANGPT2 in gastric cancer and paracancerous tissues. **(D)** Quantitative analysis of ANGPT2 in mouse subcutaneous tumor tissues. **(E)** Subcutaneous tumor samples from nude mice treated with DNAse-1 and control group, n=5. **(F)** Growth curve of subcutaneous tumor in nude mice, all values are mean ± SD. *p < 0.05 by two-way ANOVA. **(G)** Western blot analysis of ANGPT2 expression after HUVECs were stimulated by different concentrations of NETs. **(H)** Quantitative analysis of mRNA of ANGPT2. **(I)** ANGPT2 expression in HUVECs stimulated by PBS, NETs, and NETs+DNAse-1 was analyzed by immunofluorescence. **(J)** Tthe expression is indicated as the mean fluorescence intensity (MFI),analyzed with Image J software. All values are mean ± SD. ****P < 0.0001, ***P < 0.001,** P < 0.01, *P < 0.05 and ns, not significant by one-way ANOVA.

To further verify ANGPT2 expression in GC specimens, we found that ANGPT2 was highly expressed in cancer tissues from patients with GC than in para-cancerous tissues. ANGPT2 was more overexpressed in stage II/III than in stage I patients (**Figures 7A, C**). Compared with the control group, intraperitoneal injection of DNAse-1 reduced the growth volume of the subcutaneous gastric cancer tumors (**Figures 7E, F**). Staining of GC tissue derived from a subcutaneous tumor model in nude mice showed that ANGPT2 expression in DNAse-1-treated mice was significantly lower than in the control group (**Figures 7B, D**). After stimulating HUVECs with NETs, we found that ANGPT2 expression increased significantly, as evidenced by Western blot, and began to change significantly when NET concentration was 0.4 µg/ml. Consistent with our previous experimental results, DNAse-1 could significantly weaken this phenomenon (**Figures 7G, H**). Through immunofluorescence staining, it was found that ANGPT2 expression increased, and CD31 expression decreased after HUVECs were stimulated with 0.4 µg/ml NETs, while DNAse-1 could weaken the effects of NETs (**Figures 7I, J**).

## Discussion

An increasing number of studies have shown that the tumor microenvironment (TME) has important clinical significance in predicting prognosis and guiding immunotherapy. However, there is still a lack of systematic cell interaction analysis in TME. In our study, we analyzed and identified differentially expressed proteins in NET-stimulated HUVECs in GC with the help of TMT-MS/MS and PRM techniques. We found that NETs stimulation can increase ANGPT2 expression in HUVECs. Through comprehensive bioinformatic analysis, we found that the increased ANGPT2 expression is closely related to the poor survival of patients with GC. At the same time, ANGPT2 is related to the genomic changes of GC and TME. ANGPT2 can be used not only as a therapeutic indicator of immune checkpoint inhibitors but also as a target for accurate selection of chemotherapeutic drugs; thus, targeting the neutrophil/NETs-ANGPT2 axis may unveil new therapeutic targets in the future.

NETs are not only related to antimicrobial defense but also play a role in non-infectious diseases, including tumor occurrence and development, thrombosis, vasculitis, etch (28–30). Many NETs deposits have been found in the blood and tumor tissues of many solid tumors (31–33). Yang et al. found that neutrophils of patients with liver cancer, especially those with metastatic liver cancer, release more NETs; in animal experiments, they found that these NETs can capture liver cancer cells, induce cell death resistance, and enhance invasion (34). In our early studies, it has been confirmed that a large number of NETs deposit in GC tissue, significantly correlating with tumor staging. At the cellular level, NET deposits can promote the EMT process, invasion, and metastasis of GC cells, as well as the formation of a hypercoagulable state (35). In this study, we verified that NET deposition is more abundant in cancer tissues of patients with GC, accompanied by significant NET infiltration around miANGPT2cro vessels. Related studies have also confirmed that NETs can damage endothelial cells and recruit platelets to the injured site, leading to the formation of deep venous thrombosis (36, 37). This evidence shows that NETs can affect endothelial cells and trigger the onset and development of GC in the TME. Therefore, we should actively explore the specific mechanism of NETs on HUVECs. Due to the relatively low consistency between mRNA and protein expression in some tumor types, proteome analysis may outperform transcriptome analysis in detecting disease-related changes in cell activity and function (38). Therefore, in our study, proteomic technique was used to understand the changes in protein post-transcriptional translation levels after NETs stimulation of HUVECs. Thus, this approach allows us to explore the mechanism underlying the effects of NETs on HUVECs, providing a theoretical basis for the clinical treatment of GC.

Existing research results show that neovascularization in the TME provides nutrition and oxygen for tumors and is also a channel for tumor spread (39–41). Endothelial-derived SLIT2 protein and its receptor ROBO1 reportedly promote the migration and infiltration of cancer cells into endothelial tissue, whereas endothelial Slit2 knockout can inhibit tumor metastasis. In contrast, Slit2 knockout in the tumor can promote metastasis. Tumor cell-derived double-stranded RNA acts as an upstream signal to interact with RNA receptor TLR3 to induce endothelial SLIT2 expression (42). This study revealed that endothelial cells play a direct role in driving tumor metastasis and spread, proving that single genes from different cell sources can promote or inhibit cancer progression. In our study, we found that NETs act on HUVECs to trigger the release of various tumor-related factors, such as ANGPT2, ECM, CD40, and IL-8. Therefore, we have reason to believe that endothelial cells play a diverse role in the TME and can promote GC progression under the action of triggering factors.

In this study, we found that NETs promoted ANGPT2 release from HUVECs. Immunohistochemical staining showed large depositions of ANGPT2 in GC. The expression level in stage I was lower than that in stage II-III, which was positively correlated with the clinical stage, indicating that ANGPT2 overexpression occurred in the late stage of tumorigenesis. In murine subcutaneous tumor tissues, ANGPT2 expression of DNAse-1-treated mice was significantly decreased, which proved that inhibition of NETs could significantly reduce ANGPT2 expression. Consistent with this study, many studies have shown that ANGPT2 expression is significantly upregulated in breast cancer, pancreatic cancer, glioma, GC, colon cancer, liver cancer, melanoma, and other tumors. ANGPT2 plays a key role in tumor angiogenesis, tumor inflammation, and tumor metastasis (43–50). For instance, studies have shown that ANGPT2 and other factors are involved in the growth and metastasis of breast cancer, and their expression is related to the clinical stage of cancer, blood lymphatic, etc. Its high expression can lead to abnormal regulatory functions such as vascular repair and reconstruction in breast tissue, increasing the incidence of breast cancer (43). In colon cancer, an immunohistochemical study showed that the expression level of ANGPT2 protein was not related to the degree of differentiation and lymph node metastasis but to the depth of intestinal wall invasion, blood metastasis, and poor clinical prognosis (46). In normal intestinal mucosa, the expression of ANGPT2 is lower than that of intestinal adenoma, and there is a positive correlation between the angiogenic factor and tumor cell proliferation activity. The ANGPT2 expression can promote colorectal cancer growth. Therefore, the intervention of the ANGPT2 mechanism system is considered a measure to treat tumors.

## Conclusion

In this study, we used bioinformatics to predict the significance of ANGPT2 in GC. We found that ANGPT2 is a carcinogenic factor which is closely related to the tumor microenvironment in GC. It also has guiding significance with immunotherapy and chemotherapy, with a certain reference significance for clinical application.

Our method used three normal HUVECs samples and three NETs-stimulated HUVECs samples for proteomic analysis. The sample size selection was statistically significant. Our study established the relationship between NETs and HUVECs in the TME, formed a molecular network of neutrophils, NETs, and ANGPT2, and gave GC a new biomarker reference, ultimately providing a theoretical reference for the development of new therapeutic targets. These findings also support research to determine how ANGPT2-related biomarkers contribute to personalized GC treatment.

## Data availability statement

The original contributions presented in the study are included in the article/**Supplementary Material**. Further inquiries can be directed to the corresponding authors.

## Ethics statement

The studies involving human participants were reviewed and approved by this study was approved by the Internal Audit and Ethics Committee of the Second Affiliated Hospital of Harbin Medical University. The patients/participants provided their written informed consent to participate in this study. The animal study was reviewed and approved by the Internal Audit and Ethics Committee of the Second Affiliated Hospital of Harbin Medical University.

## Author contributions

SY, XZ and JL designed the study, completed the experiments, and drafted the manuscript. HY, AZ and YZ collected the patient clinical data and performed part of the experiments. LeZ and LiZ participated in the animal experiments. All authors have read and approved the final manuscript.

## Conflict of interest

The authors declare that the research was conducted in the absence of any commercial or financial relationships that could be construed as a potential conflict of interest.

## Publisher’s note

All claims expressed in this article are solely those of the authors and do not necessarily represent those of their affiliated organizations, or those of the publisher, the editors and the reviewers. Any product that may be evaluated in this article, or claim that may be made by its manufacturer, is not guaranteed or endorsed by the publisher.

## Supplementary material

Supplementary Figure 1 The genomic alterations in high-ANGPT2 and low-ANGPT2 groups. **(A, B)** Amplification and deletions in gastric cancer with high and low ANGPT2 expression. **(C, D)** The detailed amplification and deletion of chromosome copy number variation in gastric cancer with high and low ANGPT2 expression.

Supplementary Figure 2 The somatic mutations in high-ANGPT2 and low-ANGPT2groups. **(A)** The forest plot illustrates the difference in mutation patterns between high-ANGPT2 and low-ANGPT2 groups. **(B, C)** The heatmap presents the somatic interaction in gliomas with high and low ANGPT2 levels.

Supplementary Figure 3 Analysis of the correlation between ANGPT2 and other cellular immune infiltration. The lollipop illustrates the difference in immune infiltration between high-ANGPT2 and low-ANGPT2 groups.
