# Supplementary figures and images for: Immunoregulation and clinical significance of neutrophils/NETs-ANGPT2 in tumor microenvironment of gastric cancer
Source: Front Immunol. 2022 Sep 12;13:1010434. doi: 10.3389/fimmu.2022.1010434 (PMC9512293; doi:10.3389/fimmu.2022.1010434)

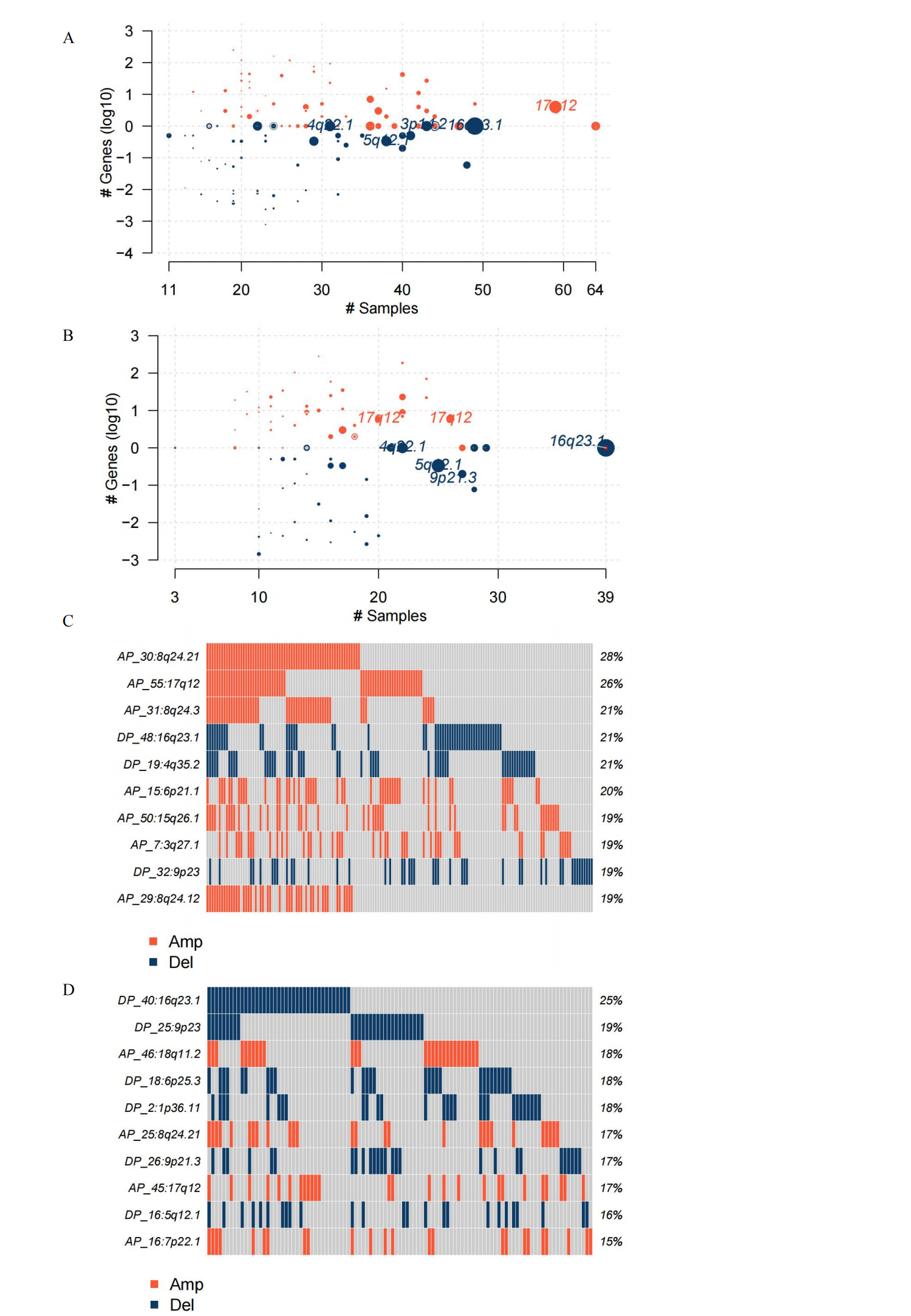

Supplement: Supplementary Figure 1 — The genomic alterations in high-ANGPT2 and low-ANGPT2 groups. (A, B) Amplification and deletions in gastric cancer with high and low ANGPT2 expression. (C, D) The detailed amplification and deletion of chromosome copy number variation in gastric cancer with high and low ANGPT2 expression. [file Image_1.tif]

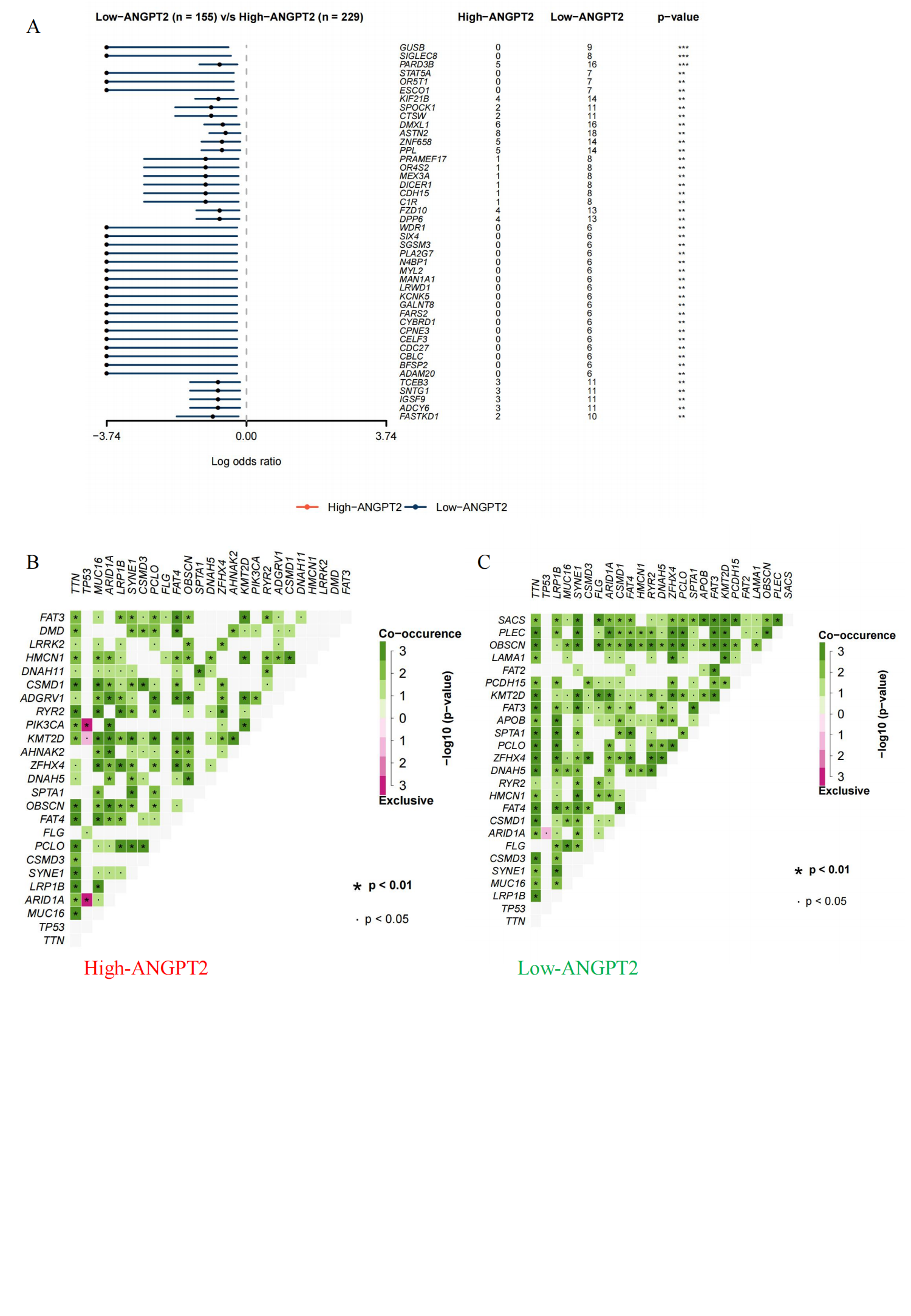

Supplement: Supplementary Figure 2 — The somatic mutations in high-ANGPT2 and low-ANGPT2groups. (A) The forest plot illustrates the difference in mutation patterns between high-ANGPT2 and low-ANGPT2 groups. (B, C) The heatmap presents the somatic interaction in gliomas with high and low ANGPT2 levels. [file Image_2.tif]

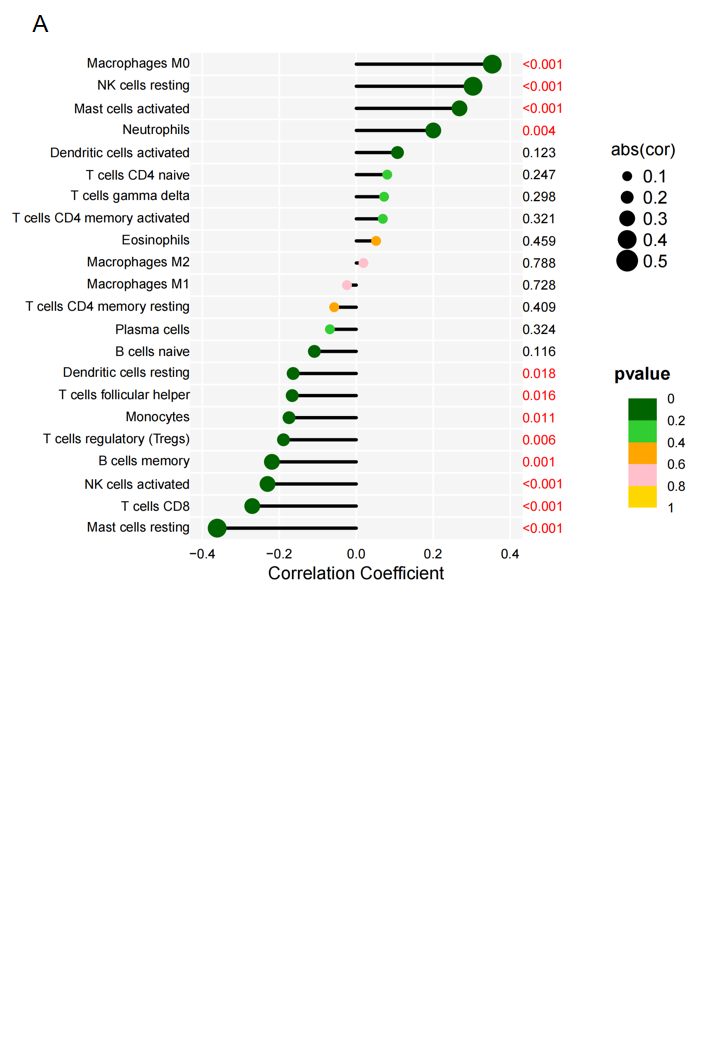

Supplement: Supplementary Figure 3 — Analysis of the correlation between ANGPT2 and other cellular immune infiltration. The lollipop illustrates the difference in immune infiltration between high-ANGPT2 and low-ANGPT2 groups. [file Image_3.tif]
